# “A torch, a rope, a belly laugh”: engaging with the multiple voices of support groups for people living with rare dementia

**DOI:** 10.3389/frdem.2024.1488025

**Published:** 2025-01-08

**Authors:** Paul M. Camic, Emma Harding, Sam Rossi-Harries, Oliver S. Hayes, Mary Pat Sullivan, Lawrence Wilson, Nikki Zimmermann, Roberta McKee-Jackson, Joshua Stott, Nick C. Fox, Catherine J. Mummery, Jonathan D. Rohrer, Jason D. Warren, Rimona S. Weil, Sebastian James Crutch

**Affiliations:** ^1^Dementia Research Centre, Research Department of Neurodegenerative Disease, UCL Queen Square Institute of Neurology, University College London, London, United Kingdom; ^2^School of Social Work, Faculty of Education and Professional Studies, Nipissing University, North Bay, ON, Canada; ^3^Independent Researcher, Sonic Studios, Rye, United Kingdom; ^4^Research Department of Clinical, Educational and Health Psychology, University College London, London, United Kingdom

**Keywords:** support groups, non-memory led dementia, young onset dementia, research poetry, linguistic analysis, thematic analysis, arts-based health research, social health

## Abstract

**Purpose:**

Rare forms of dementia bring unique difficulties related to age of onset, impact on family commitments, employment and finances, and also bring distinctive needs for support and care. The aim of the present study was to explore and better understand what the concept of support means for people living with different rare dementia (PLwRD) and their care-partners who attend ongoing support groups.

**Methods:**

Representing seven types of rare dementia, source material was collected from 177 PLwRD and care-partners attending in-person support groups, with the goal of developing research-informed group poems, co-constructed by a facilitating poet. Data were analyzed through a three-step process involving linguistic analysis followed by structured-tabular thematic analysis, relational analysis, and concluded with an online survey about participation in the study.

**Results:**

Linguistic analysis found that co-constructed poems remained faithful to the original source material offered by participants. These results provided confidence to subsequently conduct a thematic analysis of eight completed poems, identifying 15 initial themes. A further relational analysis between themes drew on six relational forms and identified an overarching theme “A Community, Not an Intervention” that describes the process of support for this population. Survey results revealed a varied but generally positive response to writing whilst reactions to the completed poems reflected strong emotional connections that resonated with personal experience.

**Conclusion:**

This is the first study that we are aware of to explore the use of co-constructed research poetry to better understand how in-person support groups provide support for people impacted by different rare dementias. The poems portray the complex, dynamic and relational aspects of how support groups provide a necessary form of connection for this population. An overarching theme characterized the support groups as a community rather than an intervention. Findings are discussed within the theoretical context of positive social identity, social health and biosocial groups. The results also demonstrate that solicited words from participants can be faithfully portrayed in poems co-created by an experienced poet. This novel finding expands methodological options for the use of research poetry in healthcare and also offers support group members further creative choices for engagement, connection and communication.

## Introduction

Over 55 million people are estimated to be living with dementia worldwide (WHO, 2020), and the condition is associated with substantial economic and social costs for individuals, families and wider society (Prince et al., 2015). The leading cause of dementia—affecting 60–70% of those diagnosed—is Alzheimer's disease, which predominantly impacts memory function and usually occurs in those over the age of 65. However, there are rarer forms of dementia which are non-memory-led, for example those predominantly affecting visual and spatial processing [as in posterior cortical atrophy (PCA), and movement and stability of attention and alertness in Lewy Body Dementia (LBD)], speech and language abilities [as in the primary progressive aphasias (PPA)], or personality and behavior [as in behavioral variant frontotemporal dementia (bvFTD)] (Crutch et al., 2017; Marshall et al., 2018; Piguet et al., 2001). There are also rarer forms of dementia which can be directly inherited [as in familial Alzheimer's disease (FAD) (Ryan et al., 2016) and familial frontotemporal dementia (fFTD) (Greaves and Rohrer, 2009)], and most of these rarer forms disproportionately affect those under the age of 65. Rarer dementias are thought to account for ~7% of all dementias (Brunnström et al., 2009; Snowden et al., 2007) and ~10–20% of dementias in people under the age of 65 (Hogan et al., 2016; Koedam et al., 2010), though these are likely underestimates due to the difficulties in getting an accurate and timely diagnosis (Kvello-Alme et al., 2019).

These conditions bring with them unique and complex challenges including difficulties related to age of onset, such as employment and related financial implications, as well as the impact on existing family care commitments (Carter, 2022; Millenaar et al., 2016). There are also challenges related to the atypicality of symptoms and widespread lack of awareness of them among health and social care professionals, friends and family, and society more broadly (Bruinsma et al., 2022; McIntyre et al., 2019; Carter et al., 2018). These include experiences of stigma, anxiety and frustration due to a lack of access to timely and appropriately tailored services (Harding et al., 2018; Roach et al., 2016; Tookey et al., 2021), increased stress and burden for carers (Karnatz et al., 2021), and isolation due to limited access to peers with a shared experience (Sullivan et al., 2022).

From healthcare and social science perspectives the use of poetry in dementia care research may seem like strange bedfellows, something that appears out of place and with low priority. Yet, over the past 20 years arts-based health research has increasingly taken an active role in dementia research alongside medical approaches that seek to cure or slow down the progress of dementia. In randomized controlled trials (e.g., Hayashi et al., 2023) and other empirical studies (e.g., Theorell, 2021), and in qualitative research (e.g., Bonds et al., 2021), the arts, including poetry, have been used “to identify and describe, understand and interpret, represent and communicate social and individual experiences of health and illness across the lifespan” (Cox and Boydell, 2016, p. 83). Ethological theories lend support to the evolutionary significance of the arts “and help to form a foundation to understand the biopsychosocial processes (Lehman et al., 2017) involved in arts participation” (Camic, 2008, p. 287). Noted anthropologist Dissanayake's (1988) research attributes the ethological importance of the arts to how they can create feelings of mutuality between people, facilitate a sense of belonging, alongside the processes of finding and making meaning.

Poetry is an artistic method (among many artistic methods) of nominating a personal (or shared) event or emotion to be appreciated by an audience that did not share that experience directly. The effect of vernacular words is heightened through techniques of, for example, rhythm, rhyme, repetition, metaphor, simile, and analogy, seeking to provide the reader or listener with insights into the poet's experience. The idea that a poem is always more than the sum of its parts allows emergent connections to be made between ideas that would not be possible via another medium. Writing, reading and hearing poetry is an embodied and interpretive experience that can generate emotional and multisensory understandings (Hanauer, 2010), whilst conveying discovery and surprise. Poetry, in casting off the syntactical rules of more typical prose forms, also manages to free itself from some of the limits imposed on language by those rules (Wenthe, 2008). In short, poetry allows for the braiding and overlapping of multiple (and sometimes contradictory) emotional truths. We can see then how poetry as a research methodology might allow for an emancipatory relationship between speaking and truth, particularly when working with groups who might otherwise struggle to get their lived experience heard (Miller, 2024). In order to incorporate the “raw words” of multiple participants into poems, the present study, as explained below, used co-constructed research poems that involved an experienced poet to facilitate this process.

Poetry as a research method can be used to represent and reinterpret existing data (Miller, 2024; Slade et al., 2023), to assemble field notes, and to collect new data (Hanauer, 2010; Kaplan and Glazner, 2023). Poetry, although distinctive from the more disimpassioned voice of usual academic discourse, allows data to be presented that has multiple meanings, is personally and inferentially constructed and allows the voice of multiple participants to be heard (Richardson, 2023), while also closely reflecting the lived experience of those impacted by rare dementia (Barnes and Warren, 2023; Wilson and Camic, 2024). The terms research poetry and poetic inquiry are sometimes used interchangeably (Vincent, 2022). For the purposes of this project, however, we were guided by the former term as a social science methodology “that involves using poetry and poetic structures to represent and analyze data” (Furman et al., 2006, p. 2). The process and experience of generating poems through research poetry can be a focus of investigation as can the completed poem itself, in addition to examining what the poem conveys about lived experience to specific audiences (e.g., healthcare practitioners, the general public, people living with a defined healthcare condition; Camic et al., 2024).

The present study, influenced by research poetry protocols outlined by Camic et al. (2022), developed poetry as a way to represent the multiple experiences of support for people living with different rare dementias who attended on-going, in-person support groups. With few exceptions, many people with young onset and rare forms of dementia have remained mostly hidden and underserved by health and social care systems in both wealthier and less wealthy countries (Sullivan et al., 2023). We specifically sought to better understand and examine, through co-constructed research poetry, how support groups provide support for PLwRD and care-partners with different types of rare dementia. A secondary aim was to interrogate the source material and completed poems using quantitative and qualitative analysis, to further develop research poetry as an arts-based health research methodology.

## Methods

This mixed-methods study involved soliciting words (source material), in order to create co-constructed poems, from PLwRD and care-partners who were participating in ongoing support groups. As an alternative to traditional qualitative research interviews, the poems created in this study were treated as data and analyzed through a three-step analytic process involving linguistic analysis followed by thematic analysis, described below. An online survey was also sent to participants along with each group's completed poems in written and audio-recorded formats. We were keenly aware that analyzing poems using social and behavioral science methodologies might seem an anathema or contradiction to the purpose of poetry as an art form. For arts-based health research to be impactful and meaningful to multiple audiences, however, both the art output (poems) and resulting analysis (linguistic and thematic analyses) are presented.

### Participants

Participants were recruited from eight support groups for people impacted by rarer forms of dementia and members of Rare Dementia Support, an organization in the London area representing seven different types of dementia (Table 1). Recruitment occurred between 4 April 2022 and 3 November 2022. Although we did not collect information about previous poetry experiences, a few participants voluntarily shared that they write and/or read poetry on a regular basis. Support group sessions occurred in accessible community settings and involved presentations from healthcare professionals on topics related to the group, facilitated discussions, informal peer-to-peer discussions and time to socially interact with peers and/or professionals (Toms et al., 2015). Seven of the support groups were diagnosis-specific and one was a mixed rare dementia group. Sixty-one percent of the 290 members [*N* = 84 (PLwRD), *N* = 182 (care-partners), *N* = 24 (people genetically at risk)] who were invited to participate took part (*N* = 177). They ranged in age from early 20s to mid-70s (Table 2) and were approximately 55% female. Ethical approval was obtained from a University College London ethics panel (approval number: 8545/004).

**Table 1:** Participation by type of rare dementia.

**Group**	***N* ^a/b^**	**Participation rate (%)**
Mixed rare dementia	14/14	100
Posterior cortical atrophy (PCA)	60/32	53.3
Familial Alzheimer's disease (fAD)	30/20	66.6
Familial frontotemporal dementia (fFTD)	11/6	54.5
Lewy body dementia (LBD)	24/18	75.0
Frontotemporal dementia (FTD) (including behavioral-variant and semantic-variant types)	56/32	57.1
Primary progressive aphasia (PPA)	66/36	54.5
Young onset Alzheimer's disease (YOAD)	29/19	65.5
Total	290/177	61.03

*N*, ^a^invited/^b^participated.

**Table 2:** Participation by age group.

**Age range**	**Number (%)**
20s	14 (5.3)
30s	26 (9.9
40s	34 (13.0)
50s	95 (36.2)
60s	80 (30.5)
70s	13 (4.9)

Informed consent was obtained from all participants involved in the study. Written informed consent was waived due to our ethics approval agreement that required recruitment of participants with only verbal consent in a group setting so as not to disrupt the activities of the support groups. The following information was presented in writing 2 weeks before each group and repeated verbally on the day of the group: “(1) that by participating in the poetry creation exercise you would be providing consent for your contribution to be included in analysis of the poetry and any possible future publications, (2) you will not be personally identifiable in any publication and, (3) that your contributions will not be linked to you or data storage or processing purposes.” Verbal consent was witnessed by one or more researchers.

### Procedure

Our approach to creating poems was designed to allow people to contribute to a poem with a single word, a series of unconnected words, or a complete sentence. We wanted to increase the accessibility of participation for people who may have specific difficulties responding due to impairment (e.g., language, memory, visual, auditory) and/or may be intimidated by writing poetry.

Approximately 2 weeks before each group met, an invitation to participate, including information about the project and ethics approval details was provided in each group's newsletter. During the support group meeting a researcher also explained the project verbally and responded to questions. Participants were given a pen and sheet of paper with the prompts written in 14-point Arial font and asked to respond in writing to either prompt; prompts were also read aloud. Responses were folded for privacy and were collected at the lunch break in order to allow time for responding.

As part of the protocol, the directions provided to participants in the newsletter and repeated at the meeting were as follows:

1. “Please write your words in any form you would like after one of the prompts. You can write one word, a complete sentence or simply any words you want to use. Since this is poetry in the making, please consider your emotions, images that come to mind, thoughts and experiences as you write.

1. How does this group support you as someone living with or living at risk of (type of dementia) or as a care-partner for someone with or living at risk of (type of dementia)?
2. How would you like the group to support you? What support do you need from this group?”

For those people who had difficulty writing or with vision issues (*N* = 3), a researcher recorded their responses and read it back to assure accuracy. Responses were anonymous and once collected were given to the facilitating poet. Using only verbatim responses the poet included words from all participants to form poems for each of the eight groups. Within 2 weeks completed poems were sent to all group members (including those who did not participate) along with a link to an online anonymous Qualtrics survey soliciting information about the process of collecting contributions (to those who participated) and reactions to the completed poems (for all members):

1. What did you think and feel about the completed poem? Please be candid.
2. If you participated in writing the poem by sharing your words, what was your experience of writing the words that you did in a group setting? Was it easy, comfortable, difficult, enjoyable, challenging, helpful/unhelpful, silly, etc.? Could we have done anything differently to make it a more useful experience?
3. In your opinion, do you think poetry writing workshop sessions, in-person or online, for people impacted by rarer forms of dementia might be helpful as a tool to help process your experiences? Please provide as much detail as you would like.
4. Do you think that reading poems like this one, created by people affected by rarer dementias, would be useful for the general public and health/social care professionals to read, in order to increase awareness of rarer forms of dementia?

### Data analysis

Data analysis involved three steps. Firstly, we investigated how faithful the completed poems were to the source material (initial responses to the prompts). To do so, linguistic analysis and word count software [Linguistic Inquiry and Word Count-22 (LIWC-22)] (Boyd et al., 2015) was used to analyze language data (Boyd et al., 2020; Kane and Van Swol, 2023). It was important to determine if the completed poems reflected the words drawn from the source material in order to feel confident that we could conduct a thematic analysis using the poems. Secondly, once this was established, a structured tabulated thematic analysis (ST-TA) (Robinson, 2022) was used to examine themes from each poem and to compare codes and themes across poems, and thirdly, this was followed by examining relationships between themes (Robinson, 2011). After each poem was sent to members, an online survey was conducted to gather free-text responses about their participation experiences. This data was also analyzed thematically.

#### Linguistic analysis

The source material and poems created from that material were compared in terms of total words, type/token ratio [total different words (types)/total words (tokens)], concreteness [the extent to which a word or concept evokes a (multi)sensory experience], age of acquisition (AoA; the age at which people learn a particular word), and contextual diversity (the number of contexts in which a word appears), plus their emotional content was examined using normative data for emotional valence (the pleasantness of a stimulus), arousal (the intensity of emotion provoked by a stimulus) and dominance (the degree of control exerted by a stimulus) (Hollis et al., 2017; Warriner et al., 2013). Wilcoxon rank sum tests (for unmatched data, though noting the lack of independence for shared words) were used for comparisons of lexical variables.

The poetry task was framed openly to encourage participation, and yielded written contributions of multiple sizes (e.g., word-level, phrase-level) and different levels of richness. These contributions were analyzed using the LIWC-22 (Boyd et al., 2022) text analysis tool. LIWC's core function is to compare words in an imputed dataset to each of its dictionaries and sub-dictionaries (>12,000 words) and assign syntactic and semantic information. For example, “howled” would register in verbs, past tense, emotion, negative emotion, sadness, and more. The global category scores for a dataset then represent the percentage of items in the dataset which fit in this dictionary. Developed by researchers interested in social, clinical, health and cognitive psychology, LIWC is uniquely placed to inform on psychological and social states identifiable at word-level. Psychological work has harnessed LIWC for 20-plus years including work on trait and personality (Chung and Pennebaker, 2014), social dominance (Kacewicz et al., 2014), group processes (Van Swol et al., 2021), and mental health research (Thompson et al., 2016), and more recently with dementia support groups (Hayes et al., 2024).

Separate files were prepared for the source material and poems for each of the eight participant groups. LIWC scores for these texts were acquired for the four standard LIWC summary variables—analytic (the degree to which people use words that suggest formal, logical, and hierarchical thinking patterns), clout (the relative social status, confidence, or leadership that people display through their writing or talking), authentic (words which reflect people talking in an honest, spontaneous way, without self-regulating or filtering what they are saying) and tone (combining positive and negative words, with higher scores indicating positive emotional tone and scores below 50 indicating negative tone)—plus more detailed affect and emotion counts (overall, positive, negative). Separate comparisons were conducted for non-corresponding words, i.e., the minority of words that appeared in the source material but not the poems, or in the poems but not the source material.

Structure within the source material and poems was considered further by generating LIWC-22 narrativity scores for staging, plot progression, and cognitive tension plus an overall measure. These scores reflect how closely any given text resembles “standard” structures used by storytellers across a range of different standard texts, with scores ranging from +100 (perfectly aligned with the normative shapes) through 0 (no relation) to −100 (opposite of normative structures). Wilcoxon signed-rank tests were used to compare source and poem structures. An additional examination of structure and word order was also piloted with one community poem, exploring the extent to which contributed words were maintained in precise form and within/outside contributed phrases in the final poem.

#### Structured tabular thematic analysis

There are several approaches to thematic analysis used in the social and healthcare sciences (Chafe, 2017; Saunders et al., 2023) with some adhering to a prescribed epistemological stance (Byrne, 2022) while others take a more pragmatic approach (Barker and Pistrang, 2021), which allows for increased analytic flexibility. Considering the present study used a mixed-methods approach that also involved many more participants than is usual in qualitative research, and the data is drawn from relatively brief texts, the structured tabular approach to thematic analysis was chosen (ST-TA) (Robinson, 2022). ST-TA partially draws upon Boyatzis' (1998) and Braun and Clarke's (2012) approaches to thematic analysis whilst underpinned by a critical realist epistemology (Bhaskar, 2016; Willis, 2023) that seeks to balance reflective and positive-reductionist approaches. Robinson's approach was also developed to analyze briefer texts than the more typical 2-h long interviews found in many qualitative studies. The Standards for Reporting Qualitative Research (O'Brien et al., 2014) guided the qualitative components of the study.

## Results

It was decidedly not our intention to subsume reading the poems with a formal analysis. We are aware that the poems themselves may portray deeper and more vivid meanings than can be identified analytically, and that is, we argue, one of the contributions of poetry and arts-based health research to portraying the human condition. However, putting these important considerations aside, this research sought to explore how research poetry might be used as a methodology to further our understanding of what support means for people impacted by rare dementia. The eight poems and source material created in this project can be found in Supplementary material S1, along with explanations of the facilitating poet's creative processes. The poems are referenced in the linguistic and thematic analyses below. They can, however, also be read independently of our analysis, which we encourage readers to do.

### Linguistic analysis

**Figure 1:**
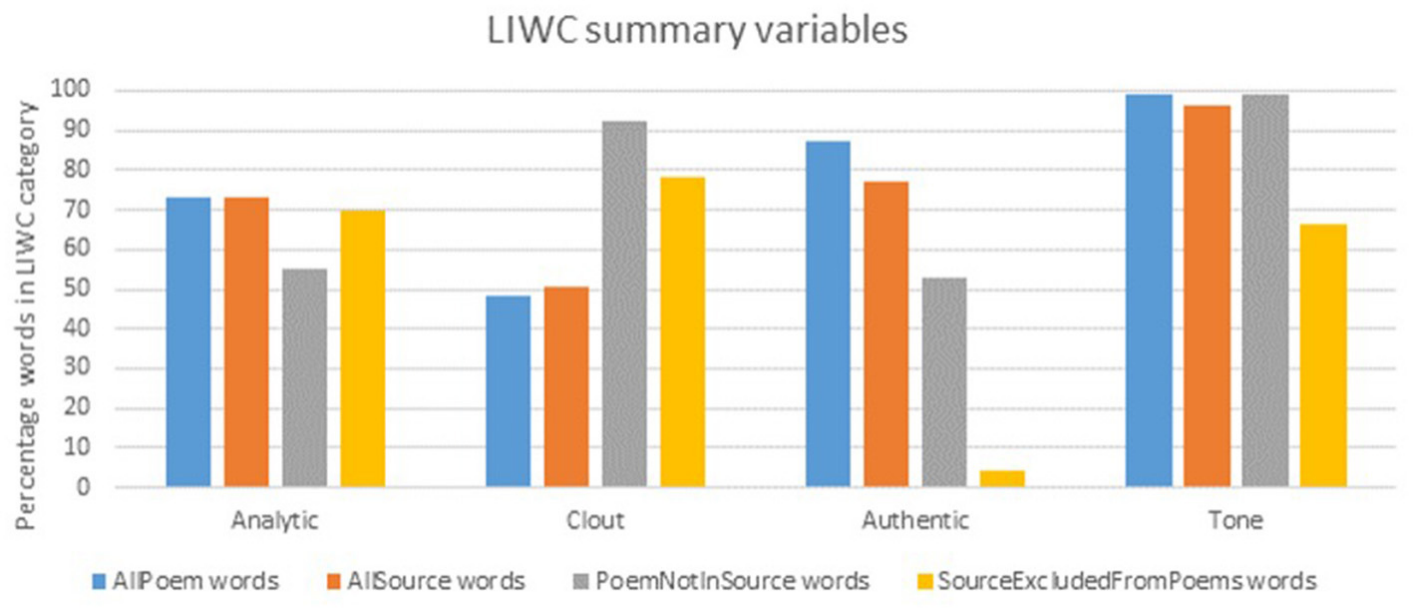
LIWC summary values across source material and poems.

**Figure 2:**
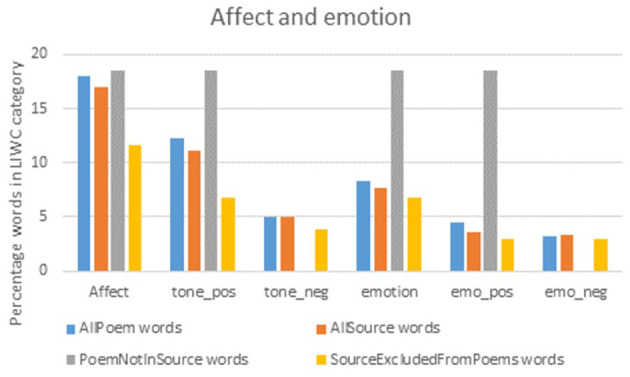
LIWC affect and emotion across source material and poems.

The source material (*N* = 1,929 words) and words used in the finished poems (*N* = 1,807) showed a high degree of correspondence, with 65% of source words appearing at least once in the corresponding poem, and 89% of poem words being contributed in the source material. No significant differences were found in any of the linguistic variables examined, suggesting that overall, the poems were faithful (i.e., well-matched) to the source material (Table 3). In the analyses of non-corresponding words (Figures 1, 2), the few source words that were not included in the poems tended to be words not classified as “authentic,” and where words with affect were added to the poems, they were slightly more likely to be positive than negative, though the number (%) of words added is very small [27 (5%)] and probably has little bearing.

**Table 3:** Comparison of source material words to completed poems.

	**Source material**	**Poems**	**Correspondence/comparison**
Total words	1,929	1,807	65% (1,253/1,929) source words in poem
			89% (1,615/1,807) poem words in source material
Total different words	663	587	84% (560/663) source words in poem
			95% (560/587) poem words in source material
Type/token ratio	0.34	0.32	–
Valence (Ns 659, 583)	0.59 (0.12)	0.59 (0.12)	*z* = −0.41, *p* = 0.69
Arousal (Ns 659, 583)	0.38 (0.06)	0.38 (0.06)	*z* = −0.40, *p* = 0.69
Dominance (Ns 659, 583)	0.58 (0.08)	0.58 (0.08)	*z* = −0.41, *p* = 0.68
Concreteness (Ns 659, 583)	0.44 (0.16)	0.44 (0.16)	*z* = 0.18, *p* = 0.85
Age of acquisition (Ns 659, 583)	7.95 (2.44)	7.89 (2.37)	*z* = 0.31, *p* = 0.75
Contextual diversity (Ns 513, 452)	8.21 (1.95)	8.27 (1.90)	*z* = −0.44, *p* = 0.66

Type/token ratio = total different words (types)/total words (tokens). Mean (SD) ratings for emotional content of words assessed using normative data for emotional valence, arousal and dominance plus concreteness, age of acquisition (AoA) and log_10_ contextual diversity (lgCD) (47–48).

Narrativity scores for each of the source materials and poems are shown in Table 4. Although poems yielded numerically higher scores for each narrativity measure except cognitive tension, there were no significant differences on these measures. The manual examination of word order used poem 4, Reminder, (fFTD; see Supplementary material, p. 10, 11 and below) as an example.

**Table 4:** LIWC-22 narrative arc scores.

	**LIWC-22 arc of narrative average score**		**Staging**		**Plot progression**		**Cognitive tension**	
	**Source**	**Poem**	**Source**	**Poem**	**Source**	**Poem**	**Source**	**Poem**
Group 1 mixed^a^	8.39	58.27	61.89	69.85	7.93	77.57	−44.87	27.38
Group 2 PCA^b^	−3.23	49.05	47.2	83.68	−20.29	−1.54	−36.58	65.02
Group 3 fAD^c^	20.47	20.74	41	44.31	−26.82	39.57	47.24	−21.65
Group 4 fFTD^d^	21.64	−25.31	67.63	−20.79	47.8	−36.58	−50.52	−18.58
Group 5 LBD^e^	−18.15	−32.38	−17.29	−19.98	1.98	−12.45	−39.12	−64.71
Group 6 FTD^f^	−39.47	2.4	−29.23	73.67	−42.03	−29.63	−47.14	−36.84
Group 7 PPA^g^	27.54	31.16	43.32	71.51	7.53	−24.41	31.77	46.38
Group 8 YOAD^h^	40.14	54.85	75.88	83.96	−19.54	75.97	64.09	4.63
*Wilcoxon signed-rank test comparisons*	*z =* –*1.26, p = 0.25*		*z =* –*1.40, p = 0.20*		*z =* –*0.70, p = 0.55*		*z =* –*0.56, p = 0.64*	

^a^Mixed forms of rare dementia, ^b^posterior cortical atrophy, ^c^familial Alzheimer's disease, ^d^familial frontotemporal dementia, ^e^Lewy body dementia, ^f^frontotemporal dementia (FTD) (including behavioral-variant and semantic-variant types), ^g^primary progressive aphasia, ^h^young onset Alzheimer's disease.

Reminder

*caring, supportive*
*insight and information*
*I am not alone*
*not alone—helpful*
*valuable understanding*
*unconditional*
*unconditionally*
*don't feel so isolated*
*understanding me*
*me, understanding*
*reassurance, connection*
*to others, caring*
*caring for others'*
*rare dementia—understanding*
*their experience*
*experiences*
*the group provides, reminds, offers*
*support, help, and care*
*caring, supportive*
*insight and information*
*reminds me—not alone*

Of the 58 individual words in this poem, 49 (84%) appeared exactly as in the source material, with the remaining 9 (16%) being adapted from source words (e.g., caring –> care). 50/58 (86%) of the individual words were drawn from phrase rather than single word source contributions. Thirty-seven words featured in the final poem as whole phrases or fragments of phrases; 33/37 (89%) of those words maintained the exact word order in which they appeared in the source material. At the contribution level, all 8/8 (100%) contributions yielded at least one word which featured in the final poem. Although contributions were not prioritized or ordered, the poem drew on each contribution in a non-sequential fashion, with the sequential order of individual poem words reflecting 26 sampling transitions throughout the poem.

The results of the linguistic analysis provides confidence that co-constructed poems created by support group members and a facilitating poet remained faithful to the source material words provided by PLwRD and care-partners. These results allowed us to subsequently conduct a thematic analysis of the completed poems, reported in the next section.

### Thematic analysis

Coding and theme development was an iterative process. In order to assure trustworthiness of the analysis (Nowell et al., 2017), three researchers independently coded the poems and through discussion, sought “interanalyst agreement to form a working intersubjective consensus” (Robinson, 2022, p. 197) rather than the statistical approach of interrater agreement. This more adaptable, albeit time-consuming method, allowed for deeper discussions and idea development. A flexible epistemological approach, critical realism (Willis, 2023), which is also relevant for brief texts (Robinson, 2022) was used to guide coding and theme development. Poems were read several times before beginning coding. Initial coding identified 41 codes across all poems. Table 5 contains a list of codes, frequency of codes across poems and occurrence in specific poems. This information is presented to increase transparency and clarity about the coding process and follows recommendations by Robinson (2022) to present code frequencies as part of a structural approach to qualitative research. Codes “Strategies and Information,” “Not Being Alone,” “Part of a Community,” “Connecting with Peers,” “Beacon of Hope,” “Transcendence,” “Dynamic Sharing,” “Nurtured,” “Professional Input” “Empathy” and “Rocky Journey” had the highest frequency within individual poems and occurred in at least half the poems. Other codes were more closely identified with poems representing specific rare dementia groups (e.g., “Discovering” (FTD, PPA, YOAD), “Empowering” (fAD, FTD, PPA, YOAD), “Reassurance” (PCA, fAD), “What about the Future?” (PCA, fAD).

**Table 5:** Initial codes, frequency and associated poems.

**Codes**	**Frequency across all poems**	**Occurrence in number of different poems**	**Name of poem associated with code^a^**
Beacon of hope	15	7	1, 2, 3, 5, 6, 7, 8
Brings people to the group	5	4	1, 5, 6, 7
Caring	3	1	4
Communication	3	2	5, 7
Connecting with peers	16	8	1, 2, 3, 4, 5, 6, 7, 8
Darkness	1	1	1
Dignity	1	1	6
Discovering	4	4	6, 7, 8
Dynamic sharing	11	5	3, 4, 6, 7, 8
Effectual actions	5	2	7, 8
Empathy	9	4	4, 5, 6, 8
Empowering	6	4	3, 6, 7, 8
Excluded	1	1	7
Gives me control	1	1	3
Happens from being in the group	7	2	5, 6
Hope	4	4	1, 3, 6, 8
Humor	4	4	1, 6, 7, 8
Included	2	1	7
Increasing understanding	4	3	3, 6, 7
Inspiration	2	2	6, 7
Mentoring	3	2	3, 6
Not always relevant	2	2	7
Not being alone	21	7	1, 2, 3, 4, 5, 6, 7
Nurtured	8	4	2, 3, 6, 7
Overwhelmed & heart-breaking	1	1	1
Part of a community	21	7	1, 2, 3, 5, 6, 7, 8
Place of refuge	9	4	1, 5, 6, 8
Possibilities	3	2	3, 7
Professional input	9	6	2, 3, 5, 6, 7, 8
Reassurance	2	2	2, 3
Recognition	5	3	3, 5, 6
oadmaps	7	2	6, 8
Rocky journey	10	4	1, 2, 6, 8
Safety	3	1	1
Self-expression	1	1	7
Sharing	5	3	1, 2, 6, 7
Strategies & information	35	8	1, 2, 3, 4, 5, 6, 7, 8
Supportive/support	8	6	3, 4, 5, 6, 7, 8
Transcendence	13	6	1, 3, 5, 6, 7, 8
Uncertainty	1	1	3
What about the future?	5	2	2, 3

^a^1, A glass half full (mixed rare dementia); 2, you are not alone (PCA); 3, instead of alone (fAD); 4, reminder (fFTD); 5, more than (LBD); 6, I honestly didn't know (FTD); 7, we help each other (PPA); 8, a beacon in the fog (YOAD).

#### Themes and narrative synthesis from individual poems

Supplementary Table 6 presents 15 primary themes from individual poems with accompanying narrative synthesis (Macdonald et al., 2023; Camic et al., 2024). The narrative synthesis is a brief snapshot of the identified themes and their relationship to each other within individual poems and was influenced by Robinson's (2011) use of relational analysis as an additional technique to aid data integration. As Supplementary Table 6 indicates, some themes were prominent in multiple groups whilst others were not. Themes were developed by carefully assessing the codes in Table 5. We looked for commonalities and differences between codes and made decisions about theme development aided, in part, by the frequencies of codes presented in Table 5. It is important to note, however, that whilst there is a good deal of thematic overlap between poems, the nuanced relationships between themes also indicates subtle and not so subtle variations and differences as seen in this excerpt from the poem, A Glass Half Full:

*A glass half full*
*Laughter in a dark space*
*Happy amongst friends*
*Share the difficult moments*
*Safety in numbers*
*A shared experience*
*An answering shout through the fog*
*Togetherness in a time of feeling alone*

For example, although the code “Strategies and Information” was identified in all eight poems, after further refinement (by integrating codes “Strategies,” “Strategies and Information,” “Effectual Actions,” “Increasing Understanding” and “Roadmaps”) it was identified as a theme that was prominent in four poems (mixed rare dementia, PCA, FTD, LBD). Likewise, as another example, the codes “Safety,” “Nurtured” and “Place of Refuge” shared similarities and analytically were combined to form the higher order theme, Place of Refuge, identified in seven poems. This is not to suggest that these themes are absent in other group poems, but they hold a more prominent position in these examples. An excerpt from the poem, I Honestly Didn't Know:

*Signposts the way along a rocky journey*
*Someone professional to talk with*
*People in the same situation help so much*
*Knowing I am not alone*
*An open door with a warm welcome*
*At a time when it felt all other doors had slammed shut*
*The strength to carry on*
*Help weathering the storm*
*A lantern of light*
*in what I know will be darkening days ahead*
*To talk to people who understand*
*A chance to share, laugh, cry and breathe*

In another example, the complex theme “Dynamic Sharing” is taken as an *in vivo* term named by a participant. It integrates the codes “Caring,” “Discovering,” “Dynamic Sharing,” and “Sharing” and is defined as an exchange of different types of support and a flexibility in responding to different levels of need among group members. Dynamic sharing is an action and is neither a linear nor a top-down approach but is seen as interactional and relational and occurs between PLwRD, care-partners and professionals. What is shared involves a range of phenomena and might include emotional support, humor, suggestions about financial help, personal stories and information about recent research. An excerpt from the poem, A Beacon in the Fog:

*A beacon of hope*
*Counting to ten*
*Without feeling exhausted*
*Lancing the pain*
*To find the joy still below*
*Help at last*
*World-class information*
*The long night's journey into day*
*Understanding what I am dealing with*
*Like an embracing warm hug*

#### Relationships between themes across poems

In addition to identifying themes pertinent to individual poems we were also interested in understanding the relationships between key overarching themes across the corpus of all eight poems (Supplementary Table 7). This involved actively exploring “whether a consensus, synthesis or unity can be found within the plurality of viewpoints” (Robinson, 2022, p. 195) voiced by our diverse group of participants. To do this we re-read the poems as a corpus of work, re-examined coding, themes and relations between themes across the poems rather than only within a poem. As stated previously, Robinson (2011) suggests articulating key relational forms to help explore the possible relationships between analytical themes. As a reminder to readers, our prompting question to participants was, How does this group support you? The relational statements in Supplementary Table 7 draw on six different relational forms in an attempt to develop a deeper understanding about the process of support groups for people impacted by rare dementia.

This phase of the analysis also identified an overarching or meta-theme, A Community, Not an Intervention. Support groups are experienced not as a clinical service, but as an “umbrella” that involves reciprocal, interactive and relational components and resources. Interventions are constructed medical and psychological processes and procedures that healthcare professionals “do” to/for patients, and there are circumstances and situations where interventions are necessary and helpful. The poetry written in the present study conveys something quite different however. Support is conceptually framed through taking place in a community that is strongly relational and contains multiple elements or themes. These include Dynamic Sharing, Strategies and Information, Part of a Community, Connecting with Peers, Professional Input, Transcendence and Effectual Actions. An underlying thread involves active involvement of members sharing information and offering psychological and social support with each other in the context of a non-hierarchical environment. People with lived experience along with professionals form the community, an important distinction from only peer-led support groups, groups facilitated by a professional or services offered by healthcare organizations.

### Survey feedback

Eighteen people completed surveys (11 care-partners, seven people living with rare dementia). Four (all care-partners) did not participate in contributing to the poems. Although fewer than we had hoped, responses provided useful information for future research and practice. However, having so few responses (11%), we chose only to do a descriptive analysis (Chafe, 2017). Complete responses to each question and the analysis are found in Supplementary material.

## Discussion

The present study sought to examine the use of co-constructed group poetry as an arts-based health research methodology to further illuminate and understand how rare dementia support groups provide a necessary form of connection for this population. Unlike many approaches to qualitative research, our intention was to solicit brief responses from as many participants as we could recruit in order to capture as broad a picture as possible of the collective experience of the support groups. We chose to explore the use of poetry because “Poems speak of the mortal condition…about the tragic and glorious issues of our lives” (Oliver, 1998, p. ix) in a way quite different from traditional research reports. Echoing Anglo-American poet Auden (1948, p. 60), “Poetry can do a hundred and one things, delight, sadden, disturb, amuse, instruct–it may express every possible shade of emotion, and describe every conceivable kind of event, but there is only one thing that all poetry must do; it must praise all it can for being and for happening.” The eight poems created in this study do indeed speak of the mortal condition and they also describe the process of seeking and finding support for forms of dementia that are often misdiagnosed, are not memory-led and likely to first occur before the age of 65. The words in these poems are the exact words of people living with these rare conditions and were put into poetic form by our experienced facilitating poet (LW) who had also previously worked with this population.

The poems, as one output from this research, stand on their own and convey similar and divergent experiences relating to rare dementia support; they can be read and listened to apart from or concurrently with a formal research paper. This allows the research-generated poems to become widely available for use as a resource to challenge stigmatizing cultural messages (Hagan and Campbell, 2021), to provide educational opportunities for healthcare professionals and students in training unfamiliar with rare dementia (Camic et al., 2024), as a reflective tool within support groups (Swinnen, 2016) and by medical practitioners and other learners (Whittle et al., 2022; Jack and Illingworth, 2023). From survey results the completed poems hold true to the lived experiences of those impacted by rare dementia and can be seen, not as representative of all PLwRD and care-partners, but as a reflective umbrella that resonated, mirrored and expanded upon personal experiences.

### Methodological and theoretical considerations

Research poetry as a methodology, while not a new approach in qualitative healthcare research, has more recently been attracting additional attention for the possibilities it offers in data collection, collaboration between participants, poets and researchers, and engagement with different audiences, including healthcare practitioners and the general public (e.g., Camic et al., 2024; Prendergast et al., 2008; Vincent, 2022). In the present study we were particularly interested in using poetry created by larger groups of people to form group poems that describe collective lived experiences. Building on recent online-based research with smaller groups of participants (Camic et al., 2024), the current project involved a larger sample across different rare dementias and did so within ongoing, in-person support groups. The difference is an important one. Utilizing research poetry within an existing, established group allows researchers, group facilitators and group members easily accessible reflective opportunities about their group experiences. A collaborative poem can act as a barometer for a group whilst at the same time respecting the privacy and anonymity of individual members and placing minimal burdens on participating.

The involvement of an experienced poet was also an important methodological consideration. The prompting question solicited words that in turn facilitated a direct contribution about one's lived experience, rather than participants' words being extracted from lengthier research interview transcripts or from clinical records (e.g., poetic transcription, Glesne, 1997) and “found poetry” (Lahman and Richard, 2014). Because researchers did not selectively choose which words to use the risk of researcher bias was reduced, if not eliminated, and, equally as important, the voices of participants remained prominent. Through the use of linguistic analysis with the source material and a completed poem prior to thematically analyzing the completed poems, confidence was established that this form of co-constructed research poetry remains faithful to the intent of group participants across all dementia groups. This methodological contribution helps to advance the rigor and validity of research poetry as arts-based health research.

The facilitating poet used different poetic forms and devices as suited the words received. Decisions were based on several factors such as assuring words were used from every participant, organizing responses into shared narratives, adopting poetic styles to fit the words (rather than altering the words to fit a style), grouping phrases according to the sounds of words and images they fashioned, paring down some of the longer responses, and use of rhythm and repetition. Responses were organized according to what “felt right.” For example, from the poem, A Glass Half Full (Supplementary material, p. 1–3), in explaining the poetic process, the poet used “six ‘painful' lines as a sort of responsive chorus” in order give “a sense of hope and determination, as well as insights into shared experiences of support.” Supplementary material 1 also contains a description of the poet's decision-making process in relation to each poem.

Additionally, this study offered an opportunity to examine how research poetry might contribute to theory development of support groups for this population. The psychological and social importance of “collective assembly”, described as psychological involvement in social experiences that provide a connection to others in a group (Gabriel et al., 2017) was evident across all groups (e.g., in the themes: Part of a Community, Connecting with Peers, Effectual Actions). Although more commonly associated with participating in social movements (Drury and Reicher, 2009) and attending cultural and sporting events (Gabriel et al., 2017), collectively coming together within rare dementia support groups helps to form a social identity (Clare et al., 2008) that serves to underpin the health and wellbeing benefits of group activity (Haslam et al., 2009) and contribute to the social health of members (Vernooij-Dassen and Jeon, 2016). The groups were seen to provide members with a sense of meaning, purpose and belonging that contribute to an individual's positive sense of social identity through, for example, creating feelings of “warmth,” “understanding,” “support,” “empowerment,” “sharing difficult moments,” and “being around people who understand.” Positive social identity (Tajfel, 1972, p.31) is the “knowledge that [we] belong to certain social groups together with some emotional and value significance to [us] of this group membership,” and is particularly important for people from marginalized groups where an adverse health condition is unknown, intractable or untreatable (Haslam et al., 2009). Supporting social relations that are “secure, safe, stable and felt to be legitimate” (e.g., Not Being Alone, A Place of Refuge, Connecting with Peers) “encourages social creativity… by rejecting prevailing negative stereotypes and labels… seeking to replace them with more positive ones” (e.g., Effectual Actions, Transcendence, A Community Not an Intervention) (Haslam et al., 2009, p. 6). Although Taylor-Rubin et al. (2020, p. 21) succinctly describes social identity as “...the cognitive mechanism that makes group behavior possible,” emotional and behavioral components were also discovered to be highly salient for our participants.

The link between social relationships and health outcomes has been historically well-documented. More recent research over the past 20 years has sought to explain how this occurs, identifying that social relationships influence health through psychosocial, behavioral and physiological mechanisms (Umberson and Karas Montez, 2010). Unfortunately, few research studies have explored social health amongst people living with dementia, likely due to an overall misconception that people diagnosed with dementia are not able to have satisfactory or worthwhile social relations. Yet seeing people living with dementia and care-partners from a social health perspective brings “attention to their needs of love, comfort, attachment, involvement, identity and meaningful occupation” (Vernooij-Dassen and Jeon, 2016, p. 702) and is a counterbalance to the more commonly held deficiency narrative about disabilities (Gray, 2009) to one of “active citizenship” characterized by new networks to support being a social citizen (Birt et al., 2017, p. 203). The overarching umbrella theme, A Community, Not an Intervention (Supplementary Table 7), highlights the contribution of positive social identity to social health and is one way to theoretically understand the value and importance of rare dementia support groups. This is particularly noteworthy considering a recent European consensus on the operationalization of social health and dementia has been proposed as a direction for future research and practice (Dröes et al., 2017). A multi-nation consensus offers new opportunities to influence ways to support community engagement, develop supportive physical and social environments and expand support programs offered in community-based meeting centers.

People with a risk factor, cluster of symptoms or a known diagnosis is a biological, not social group (Hacking, 2006). Yet, people who are part of these biological groups do come together for mutual support and form biosocial groups. The relatively recent concept of biosocial groups, based on the theory of biosociality (Rabinow, 1996, 2005) provides further context for our findings. For example, each group in our study is a neurodegenerative (biological) group. Yet, through a combination of being misdiagnosed and/or the length of time from initial symptoms to diagnosis, being younger than 65 years old at onset, lack of care pathways (Davies-Abbott et al., 2024), and few situated structures of support, “new social spaces for people living with rare dementias and care-partners' support are being cultivated” (Sullivan et al., 2023, p. 8). These relatively new biosocial groups, initially determined by a rare dementia diagnosis, have come to embody new social spaces and social networks that begin to create a sense of biosolidarity (Bradley, 2021). According to Bradley “circular looping effects of biosociality and biosolidarity demonstrate the way community activism and biosociality reproduce each other” (p. 552). Poetry co-created by biosocial group members, as occurred in the present study, has the potential to contribute to a group's solidarity and this solidarity, in turn, strengthen social relations and social identity.

### Strengths and limitations

A strength but also a limitation of the present study relates to the use of multiple authors for each poem. The poems contain many voices of similar and varied experiences of support and allowed a larger number of people to participate and contribute, yet not everyone contributing to any given poem would have experienced all that each poem conveys. This contrasts with the more usual singular poetic voice found in most poetry, including recent poems by Barnes and Warren (2023) writing about his experiences with speechlessness as a symptom of PPA. The depth of experience written by Barnes is raw, intense and personal but also conveys, reflects and resonates with others. We argue that both forms of poetry are valuable for healthcare practitioners and researchers, as well as for PLwRD, care-partners, family and friends.

A further strength was the involvement of a larger group of people than is often the case in research with qualitative components. This was made feasible, in part, by our decision to collect brief segments of data from people impacted by multiple types of rare dementia. Qualitative research need not be limited to the commonly used 1 h interview with 10–15 or so people; exploring length of data and collection formats can serve to strengthen and broaden qualitative approaches to research. A further strength of the study was the facilitating poet's use of only participants' words keeping the poems true to their lived experience. In addition, the increased accessibility of participation the method allowed is particularly important for those with atypical symptoms (e.g., predominant language impairments) and for those whose stories are largely underrepresented such as the people involved in this study, all of whom are impacted by rare dementia.

As with all qualitative components of this and other mixed-methods research, the sample is not necessarily representative of the population of people living with rare dementia nor of those attending support groups. It also did not include people in residential care or with levels of impairment that prohibited them from attending a community-based setting. People who attended support groups came from various locations across London and the United Kingdom and were able to travel to a central London location, having the time and resources to do so. The present study also purposely did not collect socioeconomic, ethnicity or level of impairment data. This would not have been possible under our ethics approval agreement that allowed us to recruit participants with only verbal consent. In order to assure the anonymity of those involved in the study and to allow us to collect data in a group setting, it was necessary to minimize identifiable information but this also presents a limitation in describing the sample.

### Implications for future research and practice

Biosocial dementia support groups, such as the eight groups involved in this study, offer possibilities for different types of mutual support, joint advocacy, and activism (Hacking, 2006). A previous study examined research-informed poetry in virtual environments (Camic et al., 2024) whereas the present study expanded on those findings by involving ongoing, community-based rare dementia support groups. The high participation rates in the present study, and generally positive response to the process of writing and responding to the poems, suggest implications for both research and practice. The use of linguistic analysis to reveal core narrative structures through text analysis (Boyd et al., 2020) has implications for further literary-based research with this population. To further document the experience of support over time, stages of the dementia journey (Hardy et al., 2024; Scott, 2022) and the group's development (Hoult et al., 2020), longitudinal research creating poems over a period time could provide insights into the nuances of group support, social identity, social health, agency, citizenship and biosolidarity. Co-constructing group poems can serve as a reflective, accessible, and non-intrusive way to contribute to the ethos, process and tasks of the group. Poetry used in research also has the potential to be a more democratic and questioning methodology, particularly when written by a group of people, “allowing the expression of subjective and perhaps sometimes even contradictory impressions from participants” (Hoult et al., 2020, p. 88). The overarching theme from the thematic analysis identified A Community, Not an Intervention as how support is experienced. Further understanding of this concept through the development of a theory of change could provide valuable insights for further research and organizational development (Mayne, 2015; Breuer et al., 2022).

## Conclusion

The flexibility of research poetry encourages methodological innovation that can involve different qualitative, quantitative and mixed-method analysis. The poems created in this study portray the complex and dynamic aspects of how support groups provide support for people living with rare dementia and care-partners. Through rigorous mixed-method analysis, multiple sub-themes and an overarching theme, A Community, Not an Intervention, were identified. Findings are discussed within the theoretical context of positive social identity, social health and biosocial groups. Methodologically, the results also confidently demonstrate that solicited words from participants can be faithfully portrayed in poems co-created by an experienced poet. This latter finding expands options for the use of research poetry co-constructed from personal experiences and also offers support group members further creative choices for engagement, connection and communication. Future research is proposed that uses research poetry to longitudinally explore biosociality, biosolidarity, agency and active citizenship for people living with rare dementia and care-partners within support groups.

## Data Availability

The original contributions presented in the study are included in the article/Supplementary material, further inquiries can be directed to the corresponding author.
