# Supplementary material for: “A torch, a rope, a belly laugh”: engaging with the multiple voices of support groups for people living with rare dementia
Source: Front Dement. 2025 Jan 8;3:1488025. doi: 10.3389/frdem.2024.1488025 (PMC11750841; doi:10.3389/frdem.2024.1488025)
Supplement: Supplementary file 1 [file Data_Sheet_1.pdf]

Supplemental file 1. Completed poems, the poet's explanation of his creative process and source material

This file contains the following information linked to the article 'Torch, rope and a belly laugh': Engaging with the multiple voices of support groups for people living with rare dementia:

1. Eight poems
2. Immediately after each poem a description of the facilitating poet's creative statement
3. The original source material for each poem

Audio files for each poem can be accessed here: (blinded for review)

Contents

Poem 1[1]: A Glass Half Full p. 1
Poem 2[2]: You Are Not Alone p. 4
Poem 3[3]: Instead of Alone p. 8
Poem 4[4]: Reminder p.10
Poem 5[5]: More Than p. 11
Poem 6[6]: I Honestly Didn't Know p. 14
Poem 7[7]: We Help Each Other p. 18
Poem 8[8]: Beacon in the Fog p. 22

A Glass Half Full (mixed rare dementia)[1], You are Not Alone (posterior cortical atrophy, PCA)[2], Instead of Alone (familial Alzheimer disease, fAD)[3], Reminder (familial frontotemporal dementia, fFTD)[4], More Than (Lewy body dementia, LBD)[5], I honestly Didn't know (frontotemporal dementia, FTD)[6], We Help Each Other (primary progressive aphasia, PPA)[7], A Beacon in the Fog (young onset Alzheimer's disease, YOAD)[8]

Poem 1:

A Glass Half Full

A glass half full
Laughter in a dark space
Happy amongst friends
Share the difficult moments

Safety in numbers
A shared experience
An answering shout through the fog
Togetherness in a time of feeling alone

Safe space. Outlet. Network

The group is like a family
Support and friendship
Learning empathy, tolerance, patience

Confusion comes
I feel numb

Meeting some good listeners
A torch, a rope, a belly laugh
Feeling less isolated
Friends. Relief. Mutual support

Open to ideas to learn and gather
How best to live with dementia
Information. Information is good
Learn how to deal with this progressive disease

Friendly, pleasant people in a nice environment
People who are going through the same situation
Not alone. Reassurance. Community
Understanding by a look

I am overwhelmed and heartbroken
Seeing my loved one suffering

Hoping to get some idea
On how best to live with dementia
A gift of independence, a way through
A normalisation of this new abnormal world

Help with opportunities, other experience
Not an empty attitude. Sharing with friends
Who have similar problems. Lack of threat
Useful information source—the best help

Getting guidance on coping mechanisms
How best to live with dementia
Togetherness in a time when you can feel alone
Hoping to learn how to deal

I feel lost
Alone

But I hope
I hope

Poet's explanation of his creative process for poem 1:

Upon first reading the group’s responses to the prompt, I had an instinctive reaction to the distinct division between the more positive words and phrases and those that expressed more pain. Every response was strong, and provided windows into understanding the lives of people living with dementia, but those six lines seemed to be asking for something more—to be heard? Not to be ignored, or side-lined? It was something of a challenge to get them all to fit into the same poem, and I read and re-read the source material for several days before beginning to work, trying to find an empathetic way to incorporate everyone’s voice effectively.

I organised the responses in quatrains (groups of four lines) according to what felt right together—experiences in common, shared narratives—and used the six ‘painful’ lines as a sort of responsive chorus. I hope it gives a sense of hope and determination, as well as insights into shared experiences of support.

Source material for poem 1:

I feel lost
Alone

Helps with opportunities
Glass half full—not have empty attitude
Sharing of problems with friends that have similar problems
Useful as an information source
Friendly, pleasant people in a nice environment

Safety in numbers
Understanding by a look
A way through

Safe space
Outlet
Network
Laugh in a dark space
Co-support/mutual support
Friends
Relief

Community
A shared experience
Togetherness in a time when you can feel alone

Learn to be empathic, tolerance, patience
Open to ideas to learn and gather
Information
Support\Friendship

Feeling less isolated

An answering short through the fog
A torch, a rope to grab, a belly laugh

A normalisation of this new abnormal world

Happy amongst friends
Gives me independences
Lack of threat

Meet people who are going through the same situation—learn how they cope
Get guidance on coping mechanisms
Share the difficult moments
Meet some good listeners

Group is like a family

I am overwhelmed and heartbroken seeing my loved one suffering with this progressive disease
I hope to learn how to deal with this condition and offer the best help

I hope to get some idea on how best to live with dementia
I feel numb

Confusion comes
Information is good
Other experience

Poem 2:

You Are Not Alone

You are not alone
Sensitive. Information. Excellent
The group has been a lifeline

Sharing experiences with others
Patience. Empathy. Understanding
Helps to reduce the fog

There is someone out there
A helping hand out of the wilderness
How to navigate in the dark

How to cope with day-to-day issues
Through the bad days. Through the good days
Comforting and informative advice

Hearing what others are going through
Warmth. Understanding. Support
I've found new friends for life

Being around people who understand

Similar experiences and challenges
Feels like a community

Learning and educating ourselves as carers
To make our daughter happy
As content as she can be

How to support our mum
Where to get help, updates
We need as much information as possible

Opportunities to meet with other carers
To hear stories of other people
That sense of connection—well-needed

Getting to know the professionals
Resisting help. Challenging. Persuade
Love them through it

Understanding the difficulties
How have they adapted?
Invaluable support and reassurance

Smoothing off my rough edges
Read Clear app. Velcro trainers. Electric cooker
It helps me to Keep On Rocking

Like-minded. Community
Other people's experiences
Talking to new people with PCA

Just knowing that the group exists
The support of those who understand
How much hard work is being done

More clarity, even if scary at times
Meet. Share. Ideas. Experiences
The group is there for you

Lovely to chat. To ask questions
What do you experience?
What helps you?

What might we come up against
As the disease progresses?
Getting to know the other sufferers

How to better understand the condition
Nice to be with like-minded people
Prompting me to be softer, a little less full-on

The group is there for you
Love them through it
You are not alone

Poet's explanation of his creative process for poem 2:

When I first saw the responses to the prompt, I was struck by three things. First, that so many people had taken part—I've never worked with the words of so many participants before, so I knew that the finished poem would need to be a longer one; second, that the underlying theme of support had been vocalized in so many interesting and varied ways; and third, that the sheer beauty of the words created a sort of song, an overall harmonious choir of language. I needed to find a structure that would do honour to the wonderful sounds of everyone's contributions as well as to the honesty of their meanings.

In organising this poem, I adapted a classical form known as a tercet, a term that comes from the Latin word for "three," in which groupings of three lines work to keep the rhythm running steadily from beginning to end. Normally a tercet would also feature an intricate rhyme scheme, but in the mid-20th century, poets began experimenting with unrhymed versions of traditional forms. Without the rhymes, the reader's ear pays a different kind of attention to the ways in which words cluster together, as well as to what happens at the breaks between stanzas.

Source Material for poem 2:

The group has been a lifeline

Always gives comforting and informative advice

Supports us with different issues like how to navigate in the dark
It's lovely to chat with everyone and discuss how to cope with day to day issues.
Ability to ask questions about what everyone experiences and what helps them.
Where to get help.
Nice to be with likeminded people

I want to learn how to support my mum and what we might come up against as the disease progresses.
We need as much information as possible.

Help through the bad days.
Help though the good days.
The support group are there for you.

It helps smooth off my rough edges and prompts me to try to be (a little(softer and less full on.

Having opportunity to meet with others to share ideas and experiences.

A helping hand out of the wilderness.

Hearing others with the same and what they are going through

Helps to reduce the fog! Helps to get more clarity, even if scary at times.

Just knowing the group exists and knowing that much hard work is being done to better understand the condition

Read Clear app: Velcro trainers. Electric cooker

There is someone out there!

Warmth. Understanding. Support.

Having the support of others who understand PCA

The group was invaluable when I needed support and reassurance!

YES support is EXCELLENT--well needed

You are not alone

Helps me to Keep On Rocking

I’ve found new friends for life

Hear stories of other people.
Like minded
Similar experiences and challenges

Talking to new people with PCA and understanding their difficulties and how they have adapted. Information.

The opportunity to meet with other carers and other people with PCA.
Feels like a community.
Sense of connection.
Being around people who understand.
An opportunity to hear about other people’s experiences.

We want to learn and educate ourselves as carers, to make our daughter as happy and content as she can be.

Information.

Information, getting to know both the professionals and the other sufferers.

Sensitive.
Love them through it.

Resisting help.

Challenging.
Persuade.

Patience
Empathy
Understanding

Updated information
Community support
Sharing experiences with others

Poem 3:

Instead of Alone

Meeting people in the same situation
Understand how others cope
Part of a wonderful tribe
First time here
Community
Listened to
A hug in the dark
Connection and support
Looking for information, for clarity
It feels like wings are wrapped around me

A sense of universality—not alone
I loved meeting people like me
Invaluable reassurance
To give me confidence
Advice. Not isolated
Empowerment
Gives me control
Building knowledge
Making connections
Explaining to others how I cope
Learning from and hearing others
Gives me the oxygen of the shared experience

Confident that I am getting the most up-to-date information
Secure in the knowledge that there are people to help
A reason to be optimistic for the future
In an otherwise bleak world
What about my kids?
Guidance
Hope
Informative
It's like a lily pad and a frog
Each day I jump on another pad

With a different perspective—different windows in a house
Learning that there is lots of improvement in Alzheimer's research

Having the opportunity to meet up with others
Provides some sense of a reason
What do I discover?
Is life over?
It's fine
Help
Support
Treatment is working
Outstanding from start to finish
Some degree of hope—mainly for my kids
I feel like I'm in a room with people instead of alone

Poet's explanation of his creative process for poem 3:

This poem is an adaptation of a form from the 1960s called a lune, so named because of the crescent shapes it creates on the page. The lines can be arranged by number of syllables, number of words, or, as in this case, by the length of the lines, as long as they're positioned longer to shorter and back again.

I chose this form for two reasons, the first being that the participants replied to the prompt with such a wide variety of wonderful responses, from single words to long phrases and sentences, some of which I could break into fragments and some which I wanted to use whole. This is something that a lune captures perfectly. The second, though, may seem a bit far-fetched, but I was struck in this case by the sheer pleasure of the sounds of the responses, and the crescents and scallops of the lune remind me of the patterns that an oscilloscope makes as it turns speech into a visible pattern.

Source Material for poem 3:

Advice. Listened to. Help. Support.

Understand how others cope and explain to others how I cope.

A hug in the dark.

Gives me the oxygen of the shared experience.

Allows connection and Support. Builds Knowledge.

To give me confidence that I am getting the most up to date information.
A sense of universality—not alone.
To give me hope/guidance by learning from and hearing others.
It's like a lily pad and a frog. Each day I jump on another pad with a different perspective (like different windows in a house). Do I discover/is life over/it's fine/what about my kids?

Knowledge empowerment, some degree of hope—mainly for my kids. Provides some sense of a reason to be optimistic for the future in an otherwise bleak world!

??? treatment is working.

Outstanding from start to finish.

I feel like I'm in a room with people instead of alone.

Secure in the knowledge that there are people to help.

Part of a wonderful tribe.

Making connections. Not isolated.

First time here. Looking for information, clarity, help.

Meet people in the same situation.

Having the opportunity to meet up with other people in the same situation.

Invaluable Reassurance

Informative. Gives you control. Community.

(She has limited speech). I loved meeting people like me. I loved learning that there is lots of improvement in Alzheimer's research.

Feels like wings wrapped around me.

Poem 4:

Reminder

caring, supportive
insight and information
I am not alone

not alone—helpful
valuable understanding
unconditional

unconditionally
don't feel so isolated
understanding me

me, understanding
reassurance, connection
to others, caring

caring for others'
rare dementia—understanding
their experience

experiences
the group provides, reminds, offers
support, help, and care

caring, supportive
insight and information
reminds me—not alone

Poet's explanation of his creative process for poem 4:

I knew immediately when I saw this set of responses to the prompt—short, sharp, clear, clean—that the finished poem would have to incorporate a good deal of repetition. Repetition is, I've always found, one of the most useful tools for bringing certain ideas to the reader's attention. When you read a word or phrase for the second or third time, you start to wonder more and more why it's important.

The result is a variation on the ancient Japanese form of the senryu, a centuries-old type of poem originating when poets would gather at parties to write collaboratively. These sorts of poems are very similar in style and form to the better-known haiku, but they deal directly with society and customs rather than using observations of the natural world to comment upon the human condition.

The short stanzas were traditionally linked, as these are here, using slight variations of the last line in one to start off the next stanza.

Source Material for poem 4:

Provide insight and information

Understanding

The group provides information, reassurance and valuable connection to others experiencing of rare dementia so I don't feel so isolated

Reminds me I'm not alone

Helpful
Caring
Supportive

RDS offers unconditional understanding

Poem 5:

More Than

Tearful, very emotional, scared what the future holds
Feeling alone and needing answers
Frustration and anxiety—eased
Now that somebody understands and knows I'm here

Show me the opportunities
Show me the respect for my words
Extinguish my fears
Connecting. Unity. Advice

Understanding the condition and my feelings
Here the gift I had, which has been taken from me
Can now be turned to poetry
A light in the darkness

Allow me to be that light
I want to see through your eyes
To meet other people in the same situation
Better to connect and understand

The words for people to work with
A break from caring with those who care
Finding out the ways to get more support
Sharing the experiences of being a partner and carer

Helpful suggestions and comments
Tips for my emotional and psychological well-being
Informal contact with professionals
Relaxing—which is greatly appreciated

There's follow-up. There's information
Serving up peer support
There's development—that's important
For people with LBD and their carers

The knowledge and the empathy
Awareness of nature and beauty
The heart. The love. The soul. The dance
Avoiding Switzerland service

Alone no longer
Confidence restored
Water, sun, trees, music
I am more than my diagnosis

Poet's explanation of his creative process for poem 5:

This was a particularly strong group of responses with which to work, and I'd like to thank the participants for their unflinching honesty and their lyrical eloquence. There

was so much beautiful language available that I was able to group phrases in quatrains (sets of four lines, a classic form of poetry in English) according to the sounds of the words and the images they fashioned.

I pared down some of the longer responses, changed a few verbs to their noun forms (and vice versa), and added some linking words, but on the whole participants' words have not been altered, only arranged. And out of the re-arrangement and new juxtapositions comes an important story, of solitude and fears replaced by a sense of positivity, due to the sharing of experiences and the respect that people have for one other.

Source Material for poem 5:

Peer support

To extinguish my fears

Advice empathy information

Avoiding
Switzerland
Service

Understanding
Helpful suggestions and comments
Follow-up
Informal contact with professionals
Information/ development

Frustration and anxiety eased now that somebody understands and knows we're here.
Alone no longer
Confidence restored

Showing opportunities and respect for words
Important to have words for people to work with and poetry allows my words
Here the gift I had, which has been taken from me can be used with poetry.

Awareness of nature, beauty
Heart love soul dance
Water sun trees music

I would like to find out on the ways how to get more support and tips for my emotional/psychological well-being as a partner of someone with LBD
Share experiences of being a partner-carer and learn for person with LBD

Connecting
Unity
Information
Knowledge

Advice

Tearful, very emotional, scared what future holds

This support group is a break from caring with those who care and to relax which is greatly appreciated

To meet other people in same situation

Feeling alone needing answers

Understanding of the condition and my feelings about it

A light in the darkness.
Support allows me to be a light.
I want to see through the eyes of a person with Lewy Bodies to better connect and understand.
I am more than my diagnosis

Poem 6:

I Honestly Didn't Know

What have other folks done to cope?
What have they found difficult?
What has helped?
I honestly didn't know

Signposts the way along a rocky journey
Someone professional to talk with
People in the same situation help so much
Knowing I am not alone

An open door with a warm welcome
At a time when it felt all other doors had slammed shut
The strength to carry on
Help weathering the storm

A lantern of light
in what I know will be darkening days ahead
To talk to people who understand
A chance to share, laugh, cry and breathe

A loving bridge that helps me glide smoothly
to live a meaningful life with my beloved hubby
educates, informs, and gives me hope
That one day we will have treatment or a cure for FTD.

A mutual ground for people undergoing such hardship

Invaluable support. Information. Reassurance
Dignity. Empathy. Mentoring. Education
All inspire me to keep going

Helping family develop knowledge and understanding of the disease
Personal examples of people's experiences, particularly carers
Knowledgeable, respectful, interested guidance and assistance
No longer on my own as a sister

The impact on relationships
The access to supportive advice
The information on available services
Not alone once meeting the support group

Not alone—a warm feeling
Connected. Belonging. Like-minded laughter
Que sera, sera, we are living with FTD
RDS is there for me, que sera, sera

I was grieving whilst living
I felt alone due to obsessive purchases
Sneaked indoors, randomly placed around the house
I now have a voice louder and more powerful than my own

Sharing knowledge and experiences
Centering. Keeping me going
There is access to and recognition from RDS
Even for those with non-direct FTD

I can talk to other carers about their experiences
There are experts available to answer questions
About FTD and caring for someone with the disease
Friendly. Connected. Nurtured. I am heard

Education worries. My husband reads poetry all the time
I'd always thought it was to do
With not remembering a story in a book
Targeted advice mitigates fear of the unknown

So this is what other folks have done to cope
So these are the things they have found difficult
What has helped? Hope. Awareness
I honestly didn't know

Poet's explanation of his creative process for poem 6:

This was one of the first opportunities I have had to work with such a large assembly of responses to the prompt. It was made especially interesting because of the direct, open language and evocative images—a signpost, a door, a lantern, a bridge—

which helped me to turn the responses into a story, one of a metaphorical journey from isolation and not-knowing to self-knowledge and recognition and strengths.

The form used for this poem is a tetrastich, which is a fancy way of saying that it’s grouped into stanzas of four lines each, and that it doesn’t rhyme. I moved words and phrases around until they sounded “right” together and added to the storyline. The title is taken from the last bit of the first stanza, a line which, by the time it is repeated at the very end, has significantly changed its meaning for the reader.

Source Material for poem 6:

Access to supportive advice
Opportunity to hear what others have done to cope
What they have found difficult, what has helped
Helping family develop knowledge and understanding of the disease
The impact on relationships

Invaluable support and to talk to people who understand

Sharing knowledge and experiences

Having a voice louder and more powerful than my own

Going, keeping me going, centring

Felt alone due to obsessive purchases sneaked indoors and randomly placed around the house

Not alone once meeting support group

Education worries

Education

I honestly don't know

Information on available services
Someone professional to talk with
My husband reads poetry all the time. I always thought it was to do with not reading to remember a story in a book!

Signposts the way along a rocky journey

Mitigates fear of the unknown
Grieving whilst living

Dignity
Empathy
Mentoring
Education

Nurturing
Targeted
Information
Advice

People in the same situation help so much

Information
Not alone
Reassurance
Hope

Inspires me to keep going

Warm feeling of mutual ground for people undergoing such hardship
Access to and recognition to support from RDS even for those with non-direct FTD (I.e. those with MS + FTD syndrome)

An open door with a warm welcome at a time when it felt all other doors had slammed shut

The group gives me the strength to carry on knowing I am not alone on this journey.

A lantern of light in what I know will be darkening days ahead

It gives me a chance to share, laugh, cry and breathe!

Help weathering the storm!

Que sera sera, we're living with FTD, RDS are there for me, que sera

informative
connected
belonging

laughter
connected
like minded
heard
friendly

Awareness of different aspects of FTD
Availability of experts to answer questions about FTD and caring for someone with FTD
Talk to other carers about their experiences

As a loving bridge that helps me glide smoothly to live a meaningful life with my beloved hubby

No longer alone as a sister

Educates, informs and gives me hope that one day we will have treatment or a cure for FTD.

Knowledgeable, respectful, interested guidance and assistance.

Personal examples of people’s experiences, particularly carers

---

Poem 7:

We Help Each Other

Friendship and understanding
A bit of sun on dark days
A calming wind

Breathing life into me and into us
A sense of togetherness
We can still laugh

Always someone there for me
It makes me feel part of a community
Travel companions on my dementia journey

Interesting
    But not 100% relevant
    Not at this stage

    Excluded
Disappointed
Frustrated

Enlightening. Comforting. Belonging
Acknowledgment and togetherness
Poetry speaks my music even when I cannot

A hot air balloon taking me to better places
Helping me to find the answers to my questions
To be better support to my father who is ging through this

Guidance and advice
Understanding the diagnosis
The gift of reliable information

I’d like to meet other partners
In a similar situation to me
with a husband or a wife with early symptoms

Zoom was amazingly helpful
But we are now cut off from information

Again

It’s rough
It’s tough
But we haven’t had enough

Information, knowledge, cheer and support
Connection, momentum, interaction, reassurance
Powerful in the face of a tsunami

Music, the sea, odd sun, birds—connect
Help me feel less stressed, less alone
Allay my fears

Background, backstep, backup
But (progressively) becoming more to the fore (frontal)
As times (temporal) change senses, emotions

I always find the line from Stevie Smith
Not waving but drowning
Most appropriate

Friendship. Inclusion. Speaking better
Reduces sense of… that feeling of being unknown
In the unknown world of PPA

Emotional support. Exchange
Sharing other people’s experience
Networking. Not feeling alone

Professional. Invaluable
Inspiring, professional, competent
Hopeful. Wonderful. Thank you for all

The intention is to learn more about the group
To assist and support starting one
We are already in the process

Thoughts hard to align
So far it doesn’t
But possibilities there

Sharing practical ideas and opportunities
Widening my knowledge
How we help each other

Poet’s explanation of his creative process for poem 7:

I found this set of words one of the more challenging ones with which I’ve worked, as the responses of the participants varied so widely—some positive, some negative,

some questioning, some seeking information. As it is always my objective not to leave out anyone's words, even if I have to change some verbs tenses or add punctuation or connectives, I struggled a bit with exactly how to structure the poem. There were rhymes, but not enough to craft a traditional rhyming form. There was evocative imagery and quotations, but just as much down-to-earth description and everyday vocabulary.

In the end I used a traditional poetic structure, one that dates back hundreds of years, and arranged the participants' lines in tercets, or groups of three—but what solved the poem for me was the keyboard's indent button. Offsetting groups of tercets from the left margin served to create opportunities for an inclusive conversation, for all voices to speak and to be heard.

Source Material for poem 7:

Connect, help me feel less stressed, less alone
Allay fears, share practical ideas
Help me to be better support to my father who is going through this

Breathing life into me/us

Music sea odd sun birds

I'd like to meet other partners in a similar situation to me with a husband/wife with early symptoms
This session has been interesting but it is not 100% relevant at this stage

Poetry speaks my music even when I cannot

Acknowledgment and togetherness

Enlightening-comforting-belonging
Hopeful-wonderful-thank you for all

Widening my knowledge

Shared experiences and reduced feeling of being in the unknown world of PPA

It makes me feel part of a community

Travel companions on my dementia journey

Reassurance
Emotional support
Sense of togetherness!

Excluded
Disappointed
Frustrated
So far it doesn't

Zoom was amazingly helpful but we are now cut off from information again

Speaking better

It’s rough, it’s tough, but we haven’t had enough; we can still laugh

To help me find the answers to my questions

Always someone there for me

Connection-Interaction

Friendship/Inclusion

Friendship and understanding

Reassurance

Sharing other people’s experience

A bit of sun on dark days
A calming wind

The intention is to learn more about the group and to assist/support starting one. We are already in the process

Professional

Invaluable

Inspiring, professional, competent

Exchange
share experiences
information
knowledge
cheer
support
I always find the time from Stevie Smith, Not Waving but Drowning. Most Appropriate!

Giving of reliable information
Not feeling alone
Networking opportunity
Guidance and advice
Understanding the diagnosis

Background, backstep, backup but (progressively) becoming more to the fore (frontal) as times (temporal) change

Connection and momentum

Reduces sense of

A hot air balloon
Taking me to better places

Senses, emotions, thoughts hard to align but possibilities there

We help each other.

Powerful in the face of a tsunami

Poem 8:

A Beacon in the Fog

A beacon of hope
Counting to ten
Without feeling exhausted

Lancing the pain
To find the joy still below

Help at last
World-class information
The long night's journey into day

Understanding what I am dealing with
Like an embracing warm hug

It's like help with the road map
Connecting the dots
Even when I cannot see them

Utterly a lifeline
For me and for us

Knowledge to navigate this complicated landscape
To know how to get assistance
Translating negative into optimism

Conversation. Dynamic sharing. Strength
Makes me feel that I belong here

Hope and empathy
Through laughter and understanding
A vision for the future

Sharing information and tips
Circling the wagons but always allowing a way in

Dispelling fear
Seriously, life's a beautiful bitch
Then you die

Knowledge is powerful
Connections are helpful
Familiar. Warm. Interesting
Group activities and friendship
Empathetic insights—helpful

Positivity. Professionalism
Dispersing the fog

Poet's explanation of his creative process for poem 8:

Thank you for writing such heartfelt words. This poem is an adaptation of a traditional Japanese form called a renga, which originated a thousand years ago as a sort of party game. Groups of poets would meet to collaborative on long poems of more than a thousand lines, taking turns with the short and long stanzas and linking the poem to an overarching-but-flexible theme, one that might change while being read, like conversations at parties do. It grew out of a shorter form called a tanka and ultimately gave birth to the well-loved type of very short poem known as a haiku.

A traditional renga would most likely focus on the natural world and the changing seasons, and would allow poets the opportunity to exhibit their individual writing skills as well as their abilities to make connections between seemingly unconnected events and emotions. It seemed a good fit for the responses from this group of participants—I felt that I could "hear" all the individual voices and this form provided a way to craft them into a single conversation.

Source Material for poem 8:

Knowledge is powerful
Vision for the future

Seriously, life's a beautiful bitch
then you die

Translating negative into optimism
Lancing the pain to find the joy still below

Counting to ten without feeling exhausted
Circling the wagons but always allowing a way in
Familiar
Warm

Interesting

Professions
World class information
A beacon of hope

Hope and empathy…through laughter and understanding

Dynamic sharing
Dispelling fear
Helpful connections

Knowledge to navigate the complicated landscape to know how to get assistance

Converse with the group
Group activities
Sharing
Friendship

The long night's journey into day

Help at last

The group understands what I am dealing with
Provides helpful insights
Is empathetic

Shares information and tips
Makes me feel that I belong here

Like an embracing warm hug

Dispersing the fog

Strength
Positive
Future

Help with the road map
Connecting the dots even when I cannot see them

Utterly a lifeline for me and us
