# Supplementary material for: “A torch, a rope, a belly laugh”: engaging with the multiple voices of support groups for people living with rare dementia
Source: Front Dement. 2025 Jan 8;3:1488025. doi: 10.3389/frdem.2024.1488025 (PMC11750841; doi:10.3389/frdem.2024.1488025)
Supplement: Supplementary file 2 [file Data_Sheet_2.pdf]

Supplemental file 2: Table 6: Themes from individual poems and accompanying narrative synthesis

Poem Title	Dementia Group	Themes	Narrative Synthesis
A Glass Half Full	Mixed	Strategies and Information<br><br>Dynamic Sharing<br><br>A Place of Refuge<br><br>Part of a Community	For this group with members living with different rare dementias, the poem highlights the necessity to share a dark space and a safe space as two aspects of what support means. This was captured by "heartbreak" plus humour, hope and happiness, which is poignantly acknowledged by the phrase, "A torch, a rope, a belly laugh". The need to express pain and share hope for the future, as aspects of support, is seen as a time to connect with self (the dark, how best to live with dementia) and also connect with others (the hope, the relief). This space is, critically, relational where connecting with peers has helped to form a community.
You Are Not Alone	Posterior cortical atrophy (PCA)	Strategies and Information<br><br>Part of a Community<br><br>Beacon of Hope	This poem conveys the strong need to obtain for oneself, and to provide to others, strategies and information about the social, medical and support needs of PCA. This occurs within the vital context of being part of a community. The group is experienced as a lifeline of safety whilst also navigating the darkness of uncertainty, "a helping hand out of the wilderness". Importantly, it is a relational community space ("like-minded people', "getting to know others", "new friends for life") that involves giving to and receiving from others "through the good days" and "through the bad days", possibly like darkness and hope as both sides of a coin.

Instead of Alone	Familial Alzheimer's disease (fAD)	Part of a Community<br><br>What About the Future?<br><br>Not Being Alone	There is an intimacy in these words that are slightly different than other poems through the physical expression (e.g., "a hug in the dark"; "it feels like wings are wrapped around me"). "I feel like I am in a room with people instead of alone" and "Part of a wonderful tribe" describes the sense of being connected with others in the group and part of a community and not an intervention. More reference, as might be anticipated due to the familial and inherited aspects of this condition, to the future was seen in this poem with respect to treatment, research and worries about children.
Reminder	Familial fronto-temporal dementia (fFTD)	Unconditionality of Support<br><br>Dynamic Sharing<br><br>Not Being Alone<br><br>Empathy<br><br>Part of a Community	Not being isolated or alone, whilst perhaps a descriptive state, was powerfully felt in this poem. Terms not seen in other poems, "unconditionality" and "unconditional", convey the importance of empathic caring for others who experience fFTD. Being part of a community that understands and cares for others through personal connections, strategies and empathy as part of dynamic caring, which alone with unconditionality of support appear to be the overarching themes in this poem.
More Than	Lewy body dementia (LBD)	A Beacon of Hope<br><br>Transcendence<br><br>What About the Future?<br><br>Strategies & Information<br><br>Connecting with Peers<br><br>Professional Input	The life turbulence that brings people to this support group includes being scared, feeling alone, a tremendous uncertainty about the future and making decisions about "Switzerland service", a reference to assisted dying that is not yet legal in the United Kingdom. Together with emotional needs, people attend to pursue information and answers to questions about Lewy body dementia that have often been elusive or not available from non-specialist healthcare professionals. These questions were met by support that included recognition, hands-on strategies offered by group members during the experience of connecting with each other, and a sense of transcendence of being "more than my diagnosis".

I Honestly Didn’t Know	Frontotemporal dementia (FTD)	A Rocky Journey<br><br>A Place of Refuge<br><br>Dynamic Sharing<br><br>Professional Input<br><br>Empathy	The poem points to a rocky journey and the impact on relationships that FTD can have. The group as a “mutual ground” is seen as a place of refuge and a “loving bridge” to share how others have coped with FTD. Dynamic sharing involves members helping each other to obtain guidance, develop knowledge, gain awareness and feel strong emotional support. This empowering support helps to centre and inspire, “to keep me going” through anticipated dark days ahead and “weathering the storm” of uncertainty and isolation. Whilst support from professionals is positively acknowledged, the peer support from group members is repeatedly highlighted. Members feel recognition and empathy from and towards each other.
We Help Each Other	Primary progressive aphasia (PPA)	Dynamic Sharing<br><br>Part of a Community<br><br>Exclusion, Frustration and Disappointment	Being part of a larger, supportive and inspiring community features vibrantly in this poem. The sense that someone is always there who can provide comfort, inspiration and humour helps to balance out the roughness of the “tsunami” that living with PPA can entail. The transcendence of “A hot air balloon that can take me to better places” through “information, knowledge, kindness, reassurance” is part of dynamic sharing and is a powerful component of the group. A decrease in verbal communication abilities is one of the symptoms of more advanced PPA yet “poetry” which “speaks my music even when I cannot”, was seen as an empowering and a less difficult tool for self-expression and one that allows participation in a community. For some, however, frustration and disappointment was experienced, as was a sense of being excluded. This was an important reminder for us of communication differences, “Thoughts hard to align" need to be continually addressed, and that support is not necessarily immediate, “So far it doesn’t. But possibilities there.”

A Beacon in the Fog	Young onset Alzheimer's disease (YOAD)	Effectual Actions<br><br>Dynamic Sharing<br><br>A Place of Refuge<br><br>Part of a Community	By providing opportunities for sharing one's lived experience, together with professional expertise within an emotionally rich and empathic environment, the group offers refuge from the uncertainty and general lack of information surrounding young onset Alzheimer's disease. The group also offers effectual actions that help to mitigate uncertainty. As a perceived lifeline in times of uncertainty, the support provided by the group is experienced as being dynamic in that it emboldens members and attending professionals to connect with each other through shared stories, questioning and offering insights. These experiences help to navigate a complicated personal and healthcare landscape, cutting through deep uncertainty, dissipating fear and providing a vision for the future.
