# Supplementary material for: “A torch, a rope, a belly laugh”: engaging with the multiple voices of support groups for people living with rare dementia
Source: Front Dement. 2025 Jan 8;3:1488025. doi: 10.3389/frdem.2024.1488025 (PMC11750841; doi:10.3389/frdem.2024.1488025)
Supplement: Supplementary file 3 [file Data_Sheet_3.pdf]

Supplemental File 3: Table 7. Summary of key relational forms, thematic elements and relational statements

**Key relational form**<br>(Robinson, 2011, p. 201-205)	**Thematic elements**	**Relational statement**
Comparative relations<br>Comparative relations are those that link two or more themes by highlighting a similarity or difference between them (Glucksberg and Keysar, 1990).	Rocky Journey<br>A Place of Refuge<br>Not Being Alone	On the often long and Rocky Journey (e.g., “it’s rough, it’s tough”, “resisting help. “Challenging”. “Persuade”) from first worrisome symptoms, to obtaining a diagnosis, to finding support, the groups provide a personal safe haven that softens the sense of being alone and isolated (e.g., “In what I know will be darkening days ahead”). Even with support, it is not as if the journey does not remain difficult and challenging, but the dreaded sense of isolation is lessened (e.g., “I feel like I am in a room with people instead of alone”, “I am not alone”, “reduces the sense of that feeling of being unknown.”).
Semiotic relations<br>One theme signifies or symbolises one or more themes. Symbols have a visual or sensory resemblance to which it symbolises, while a sign has no physical resemblance to that which it signifies (Robinson, 2011).	Part of a Community<br>Connecting with Peers<br>Beacon of Hope	Being part of a community (e.g., “I’ve found new friends for life”, “share the difficult moments”, “being around people who understand”), is a sign that meaningful connections are occurring between group members. These connections with peers (e.g., “Provides some sense of reason”, “Friends. Relief. Mutual support.”, “A torch, a rope, a belly laugh”) are symbols that “provides a vision for the future”, “a light of what might be possible”, “an answering shout through the fog”.
Evocative relations<br>Linking one theme to another using a metaphor or simile (Roberts and Kreuz, 1994). “Metaphors and similes imply the nonliteral equivalence of different phenomena rather than literal linkage of the comparative relation” (Robinson, 2011, p. 202).	What about the Future?<br>Transcendence	The certainty of uncertainty is constantly present for many people with rare dementia (e.g., “What do I discover?”, “Is life over?”) and feelings of being “Excluded”, “Disappointed” and “Frustrated” are not unusual. Yet these very present life-impacting uncertainties can produce remarkable responses through group support (e.g., “A gift of independence, a way through”, “A normalisation of this new abnormal world”). A tremendous loss can be transcended (e.g., “Here the gift I had, which has been taken from me can now be turned to poetry. A light in the darkness.”)

Contingency relations<br><br>A likely or necessary condition for a phenomenon to occur.	A Rocky Journey→<br>A Place of Refuge<br>Part of a Community	The support groups provide refuge (e.g., “An open door with a warm welcome”, “Centring, keep me going”, "Understanding by a look”) from the many challenges of living with rare dementia (e.g., “What about my kids?”, “What might we come up against?”, “Is life over?”) . Although it may appear counterintuitive, the tremendous uncertainty of the journey creates the need for refuge. Refuge, however, does not necessarily imply a community. A place of refuge within the context of rare dementia, is a precursor to being part of something larger (e.g., “The group is like a family”, “Friends, relief, mutual support”, “The group is there for you”).
Reciprocal relations<br><br>Bi-directional forms of influence within social interactions.	A Place of Refuge<br>Dynamic Sharing	Feeling the “Warmth. Understanding. Support”, “Empowerment”, “Confidence” of a place of refuge is influenced by, and also influences, multiple forms and types of sharing (e.g., “Sharing practical ideas and opportunities”, “The group reminds, provides, offers support, help and care”, “There are experts available to answer questions”, “Connecting the dots even when I cannot see them”).
Conceptual part-whole relations<br><br>Multiple lower-order themes are linked to an overarching, higher order conceptual theme.	A Community, Not an Intervention<br>~Dynamic Sharing<br>~Strategies and Information<br>~Part of a Community<br>~Connecting with Peers<br>~Professional Input<br>~Transcendence<br>~Effectual Actions	Although the members of the support groups seek strategies, information and help from each other and professionals that attend, the relationships between professionals and PLwRD and care-partners are reciprocal, interactive and relational. Professionals are not described as providing an intervention, which is more typically associated with their role. Connecting with Peers (e.g., “To hear stories of other people”, “That sense of connection-well needed”, “I loved meeting people like me”) is a vital component of support and involves physical and emotional interaction. PLwRD and care-partners along with professionals, contribute to the group through what we have identified as Dynamic Sharing (e.g., “Through laughter and understanding”, “Information of available services”, “Translating negative into optimism”, “New research”) . The support felt from the groups as a

		community provides a sense of self Transcendence (e.g., “It’s like a lily pad and a frog. Everyday I jump on another pad”, “Dispersing the fog", "A hot air balloon taking me to different places”), Effectual Actions (e.g., “Dispelling fear”, “Lancing the pain”), Connecting with Peers (e.g., “I loved meeting people like me”, “Caring for others”).
