# Supplementary material for: “A torch, a rope, a belly laugh”: engaging with the multiple voices of support groups for people living with rare dementia
Source: Front Dement. 2025 Jan 8;3:1488025. doi: 10.3389/frdem.2024.1488025 (PMC11750841; doi:10.3389/frdem.2024.1488025)
Supplement: Supplementary file 4 [file Data_Sheet_4.pdf]

Supplemental file 4. Survey Responses: Descriptive qualitative analysis followed by verbatim responses to questions

The experience of contributing to the poems varied but was generally positive and at times challenging for those living with dementia (e.g., “Thought provoking, reflective and hard” (PLwRD, PPA), “Easy to do but the words were hard to write (PLwRD, fAD), “It was a little challenging but challenges can be good” (PLwRD, PPA), “Easy and comfortable to do. Kind of exciting as I have never done this before” (PLwRD, YOAD), “Never did this before. Challenging but useful and interesting” (PLwRD, PCA)). Two care-partners (C-P) noted there could have been more time to write, (e.g., “I wish we had a little more time. Maybe given 10 minutes just before going to lunch to write (C-P, PCA), “I felt a little rushed” (C-P, fAD)) and one noted a lack of privacy, “We didn’t have the space to privately write our contributions” (C-P, fAD). This did not appear to detract from the overall positive experience, however (e.g., “I was surprised how our words could be brought together so well and how the poet captured real life. I liked doing it in the group. Powerful.” (C-P, PCA), “I don’t write poetry but this was helpful” (C-P, svFTD), “Any opportunity I get to share my thoughts is a wonderful bonus” (C-P, bvFTD).

Responses to the completed poems reflected a strong emotional connection that was “a mirror and a beacon” (C-P, PCA) as they “hit the mark” (PLwRD, LBD) by portraying how groups support PLwRD and care-partners. In reflecting lived experience, participants described a range of reactions to the poems including contrasting the poem to the disease (e.g., “It gave me a feeling of peace. The reading was beautiful and it gave beauty to an ugly disease” (C-P, fAD). Reading was also “heart wrenching but at the same time like a mirror to my life these past few years. I have re-read it and listened to it several times and find it the first true description of our experiences” (PLwRD, fAD). In commenting about listening (in contrast to reading) to the poem, “It perfectly explains the diversity of emotions we experience when living with this disease…..especially when read aloud.” (C-P, bvFTD). Considering what the poems might provide to this population, “The mix of words opens new thoughts on our experiences” (PLwRD, PPA), “...it is a beautiful way to convey how people feel without the raw emotion or shyness to explain thoughts” (C-P, fAD), and “I think it could help the 'carers' of someone with a rare dementia to allow themselves the time and opportunity to put into written words the many emotions and thoughts that they often don't allow themselves to feel and think “(C-P, PPA).

All but one survey response positively endorsed that poetry could be used as a tool to increase awareness of rare dementia by the general public and healthcare professionals with four participants planning to send poems to GPs (e.g., “Poems could be a very powerful way of helping to increase awareness and insight” (C-P, PPA), “Poetry is one form of information and …alongside lectures and clinical/technical information would help personalise and humanise the condition. I'm sending it to my GP.” (PLwRD, LBD).

Participant (Group membership)	Q1: Experience of writing	Q2: Response to completed poem	Q:3 Poetry writing workshops as a tool to process your personal experiences	Q:4 Poetry as a tool to increase awareness of rare dementia by the general public and health/social care professionals
1 Care-partner (PCA[1])	I wish we had a little more time. Maybe given 10 minutes just before going to lunch to write. I was surprised how our words could be brought together so well and how the poet captured real life. I liked doing it in the group. Powerful.	It captured my life. A mirror and a beacon.	They could if we had time to do it in the group or maybe online too. It's another way to process and make sense of things that don't make sense.	Yes. Yes. Yes.
2 Care-partner (fAD[2])	I felt a little rushed as others I was with were leaving for a tea break.	I felt the poem was an insight to all the different ways people feel in such a difficult situation. It really resonated with me.	I’m unsure as I’ve never done anything like this before.	Yes I think it’s a good way to talk about the different emotions & problems that individuals face in such life changing times
3 Care-partner (fAD[2])	It felt uncomfortable but I’m glad I did I it. We didn’t have the space to privately write our contributions.	It gave me a feeling of peace. The reading was beautiful and it gave beauty to an ugly disease. It gave me a real sense of what the room was feeling when in a seminar forum the audience is very silent. I was dubious about the impact of my words but I’m so glad I participated.	Yes, it is a beautiful way to convey how people feel without the raw emotion or shyness to explain thoughts	Yes, definitely they could be used as a soundtrack to adverts or something similar. Hearing them read out is more powerful than assuming people will read them. You immerse yourself in the words

4 PLwRD (fAD[2])	Easy to do but the words, well the words were hard to write. The professor who explained it plus the introduction we were sent beforehand was clear and I knew immediately I wanted to participate.	It was heart wrenching but at the same time like a mirror to my life these past few years. I have re-read it and listened to it several times and find it the first true description of our experiences. It was good to see my words among the many. Very special.	Not sure. I liked that this was done as a group exercise and the professional poet's skill and sensitivity came through.	Absolutely. I am sending this to our GP and consultant.
5 Care-partner (PPA[3])	I was not one of the writers.	I found it moving - and unexpected at times.	I think it could help the 'carers' of someone with a rare dementia to allow themselves the time and opportunity to put into written words the many emotions and thoughts that they often don't all ow themselves to feel and think.	I do. So little is known about the rare dementias even within many of the healthcare professions let alone the general public. Poems could be a very powerful way of helping to increase awareness and insight.
6 Care-partner (svFTD[4])	I don't write poetry but this was helpful.	Reassured. It did feel like the poem enabled a lot of aspects of rarer dementias, (both for those with dementia, and carers I think, but I can only really speak from my perspective as a Carer really). My mum has FTD, & I don’t think she would have ever ‘got’ it, but maybe that’s because she has semantic variant.	I don’t really feel like poetry is for me. Sometimes I like some poems, but I can find myself thinking that poetry is a bit boring/something that doesn’t interest me. Occasionally I like the idea of spoken word (I guess the idea of a work being performed helps).	It conveyed many aspects of rare dementia.

7 Care-partner (bvFTD[5])	Hardly need to respond really…it is probably quite apparent that I LOVE words and am happy to share with anyone who will listen! FTD has shrunk our horizons (my husband and I) almost to non-existence, which is not a problem in itself: we have learned to deal with it, but we do not talk much to anyone else. Any opportunity I get to share my thoughts is a wonderful bonus.	Completely faithful to the words and phrases given. It perfectly explains the diversity of emotions we experience when living with this disease…..especially when read aloud.	Absolutely! Writing down one's thoughts is a powerful tool….to see them created into something beautiful by the poet is magical. Thank you so much. People are often scared of poetry and this would be an ideal way of helping them to embrace it. To get comfort from it. In my experience, all poetry is driven by the passions of the creator and you couldn't find a more passionate group than those connected to FTD.	Certainly…and, as I have said, hearing them read aloud gives them a depth of meaning and import, which is missing in one's own head, which in turn is informed only by one's own experience.
8 PLwRD (PPA[3])	Thought provoking, reflective and hard. Maybe try offering lots of optional words to choose from as well as spontaneous ones.	Another source of reassurance that our experience is normal, seems genuine and a reality check.	Yes the mix of words opens new thoughts on our experiences and shares feelings in an anonymous way.	Yes but might need more direction to give more specific direction for learning, maybe think about same approach for the professionals to share their experiences too.
9 Care-partner (bvFTD[5])	I think by writing poetry myself helped me massively going through this as a main carer, I could put down in words exactly how I felt, be angry, or sad. For my husband as a sufferer it would definitely not be his thing. I usually write my poems at night when my thoughts come into my head.	For me personally I thought the voice sounded computerised therefore lacked feeling, it was far too long, the singing was dated, I say that because the majority of FTD patients are usually under 65.	Personally I could not write in a group setting. Poetry has been a powerful tool for me over the years, expressing how you feel about dementia and the effects it has on your life has been the best therapy.	No, the barriers I have come across and the battles I have fought for my own husband to get the correct care even whilst in a hospital setting, unless you are in it if you read a poem it would mean nothing<br>I have been his carer now for 7 years, just me & him behavioural FTD.

10 Care-partner (LBD[6])	NA	It was very powerful. It gives an insight into the different feelings following a diagnosis and the things that help the person with LBD and their supporters. Some things are not easy to hear but need to be heard. If someone is saying it then they are feeling it.	Yes . I believe capturing someone's words on paper gives an opportunity for that person and those involved with them to explore and reflect on what is said. Creating poetry is positive. There is an end product to be celebrated.	Absolutely. Nothing is more educational than the experience and words of those who live with the condition whether it be a person with the condition or someone supporting /caring for them.
11 Care-partner (bvFTD[5])	I did not participate in this particular group activity, but was involved in the earlier on-line group. I imagine it was all of the above things and I would look for those indicators of involvement as being incredibly positive: reflecting the diversity of life….especially one dominated by the horrors of a rare dementia.	Absolutely to the point. Many colours and stages of awareness - all inclusive. Apposite.	For Carers, yes. Unlikely to be continuously accessible to those with bvFTD (our experience) as too much concentration leads to overload, headache and enforced sleep. Barriers in perception of what Poetry is; can I do it!	Yes, very much so. I believe it is essential to present this audibly, as people are not likely to access text readily, as mentioned above. The poem comes 'alive' when read out. There is a particular intimacy then about the language selected by the authors.
12 Care-partner (PPA[3])	NA	A bit diverse/hopscotch - but accurate.<br>Cos that's how it's felt as a carer for someone with PPA - one word of uplift and the next utterly blocked.	100% yes.<br>My only experience is caring for my Mum with lvPPA and every word left is sacred. We took part in the Creativity Club 2021, last year? And when it came to poem writing I assumed it was for those with PCA only . I was utterly wrong - she managed a simple and clear poem. And yet again I realised just how capable she still was (and I think I am reasonably tuned in!)	Yes. It's seen as such a grim subject, full of pity and casting the person aside.<br>No one wants to read a fact sheet on it. The huge majority of people think of their nan wandering streets in nightie with poop stains.

13 PLwRD (LBD[6])	Worked very easily. The chap (forget his name) was clear and helpful.	It hit the mark and made a lot of emotional sense to us.	Yes. I had not appreciated this might be the case but having someone help me/us to contribute to poetry in the way it happened in the group would be something to try.	Poetry is one form of information and it could be possible that poems, alongside lectures and clinical/technical information would help personalise and humanise the condition. I'm sending it to my GP.
14 PLwRD (PPA[3])	It was a little challenging but challenges can be good. I had no difficulty with it and appreciated being asked to take part.	It gave a range of experiences, many of which I could identify with. It helped to provide a different perspective of PPA---emotional, honest, kind.	Yes if they are kept brief (1 hour) for 1 to 3 meetings, and focused. There would need to be a topic and time to explore variations.	Absolutely. Might be hard to read but takes less time than a news story and possibly more hard hitting.
15 Care-partner (YOAD[7])	No problems at all. Getting the questions ahead of the meeting helped us to think about them.	Emotionally strong and meaningful.	I think they could be useful. Not sure how they would be run but online would be easier. Perhaps a few sessions over a month? Longer? I'm not sure.	Anything to increase awareness, please. Even if just 10 health professionals read it, that would be 10 more.
16 PLwRD (YOAD[7])	Easy and comfortable to do. Kind of exciting as I have never done this before.	It made me cry. Amazing how a collection of words can be put together so eloquently from such a large group.	I have never written poetry so not sure. We'd need an experienced poet to help out.	Yes! Put them in consultants' waiting rooms. Send them to GPs. In newspapers too.
17 PLwRD (fFTD[8])	A little difficult because coordination is sometimes hard to do but I liked the idea of doing it in a group. I wish we could have discussed it.	That's my world. A good reflection of "living" as I do. Talented poet.	If they are too complex it would be hard for me. I live alone and sometimes the computer is confusing. In person is better.	Yes. My GP still thinks I have old people dementia so he needs to see them. I can do that.
18 PLwRD (PCA[1])	Never did this before. Challenging but useful and interesting. Wish we had the opportunity to discuss the poem.	It spoke to me and my condition. Emotionally charged, sensitive, encompassing.	Yes it could in a group situation where we had time to talk with the poet too.	Absolutely. I will send this to my GP.

[1] posterior cortical atrophy, [2] familial Alzheimer's disease, [3] primary progressive aphasia, [4] semantic variant frontotemporal dementia, [5] behavioral variant frontotemporal dementia, [6] Lewy body dementia, [7] young onset Alzheimer's disease, [8] familiar frontotemporal dementia
